# Pitted keratolysis

**DOI:** 10.11604/pamj.2022.41.289.26065

**Published:** 2022-04-08

**Authors:** Raghul Saravanan, Krishna Prasanth Baalann

**Affiliations:** 1 Department of Community Medicine, Sree Balaji Medical College and Hospital, Bharath Institute of Higher Education and Research Institute, Chennai, Tamil Nadu, India

**Keywords:** Foot hygiene, palmoplantar hyperhidrosis, anti-perspirant

## Image in medicine

Pitted Keratolysis is a descriptive title for a superficial bacterial skin infection that affects the soles of the foot, less frequently, the palms confined to the stratum corneum. The etiology is often attributes due to Kytococcus sedentarius and Corynebacterium species bacteria. Pitted keratolysis is most common in the age group of 21 to 30 years, with a majority of affected patients in their 1^st^ to 4^th^ decade of life. Males are at 4 times higher risk of being susceptible to this condition, presumably, due to frequent use of occlusive footwear, whereas females maintain better foot hygiene. We present a case of a 23-year-old medical intern who presented to our hospital with complaints of pitted skin lesion over base of foot, predominantly over toes for past 3 days. Patient had 3 duty days in the previous week where he wore shoes for about 15 hours continuously. Patient also gave history of excessive sweating of palms and soles since childhood. On examination pitted lesions were noted and pungent odor emanated from the foot (A). Patient was suggested to clean and dry the foot and apply Mupirocin ointment for one week. Patient was advised to use anti-perspirant and change socks twice a day. Patient showed significant improvement on follow up after 2 weeks (B).

**Figure 1:**
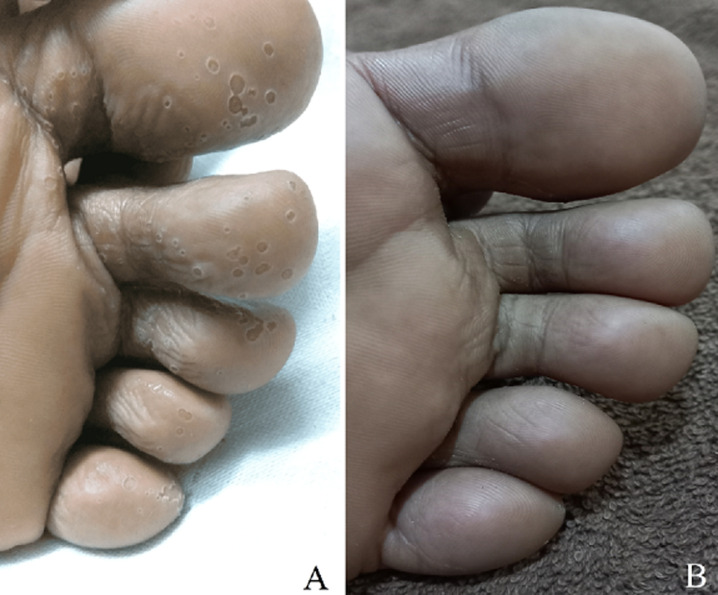
pitted lesions noted in sole of foot before treatment (A), disappearance of lesion after treatment (B)

